# Quality-of-Life Changes Following Cisplatin-Based Chemotherapy in Patients with Lung Cancer and Cisplatin-Induced Peripheral Neuropathy: A Sex-Stratified Analysis

**DOI:** 10.3390/cancers18213515

**Published:** 2026-11-01

**Authors:** Sanja Tomanovic Vujadinovic, Nevena Krstic, Ivan Selakovic, Milica Aleksic, Aleksandra Vidakovic, Emilija Dubljanin, Dragana Jovanovic, Nela Ilic

**Affiliations:** 1 Faculty of Medicine, University of Belgrade, 11000 Belgrade, Serbia; drnevenakrstic@gmail.com (N.K.); ivan.selakovic@gmail.com (I.S.); aleksandra.vidakovic@med.bg.ac.rs (A.V.); edubljaninraspopovic@gmail.com (E.D.); nelavilic@gmail.com (N.I.); 2 Center for Physical Medicine and Rehabilitation, University Clinical Centre of Serbia, 11000 Belgrade, Serbia; aleksic.s.milica@gmail.com; 3 Clinic for Rehabilitation “Dr. Miroslav Zotovic”, 11000 Belgrade, Serbia; 4 Meliora Medica, 11000 Belgrade, Serbia; draganajv@yahoo.com

**Keywords:** lung cancer, cisplatin, chemotherapy-induced peripheral neuropathy, quality of life, sex differences, EORTC QLQ-C30, FACT-L

## Abstract

**Background/Objectives**: Peripheral neuropathy is an important neurotoxic complication of cisplatin-based chemotherapy, but its occurrence may be accompanied by different changes across specific aspects of quality of life (QoL). This study aimed to evaluate longitudinal QoL changes in patients with lung cancer and determine whether they differed between women and men. **Methods**: This longitudinal analysis included 40 patients with lung cancer who developed cisplatin-induced peripheral neuropathy during cisplatin-based chemotherapy. QoL was assessed before chemotherapy and after four cycles using the European Organisation for Research and Treatment of Cancer Quality of Life Questionnaire Core 30 (EORTC QLQ-C30) and Functional Assessment of Cancer Therapy–Lung (FACT-L). Changes over time, sex differences, and time × sex interactions were evaluated using a repeated-measures General Linear Model. *p*-values were adjusted for multiple comparisons using the Benjamini–Hochberg procedure. **Results**: Of 40 patients, 27 (67.5%) were men and 13 (32.5%) were women. On the EORTC QLQ-C30, no significant time effects were observed after adjustment for multiple comparisons. On the FACT-L, Social/Family Well-Being decreased significantly (FDR-adjusted *p* = 0.024), whereas Lung Cancer Subscale scores increased significantly (FDR-adjusted *p* = 0.044). No significant main effects of sex were identified after adjustment for multiple comparisons. No significant time × sex interactions were identified for any outcome. **Conclusions**: Significant changes were observed in selected QoL outcomes during cisplatin-based chemotherapy, while no significant sex-related differences in longitudinal changes were identified. Given the small and sex-imbalanced sample, the absence of significant interactions should not be interpreted as evidence of equivalent QoL changes between women and men.

## Simple Summary

Cisplatin is widely used to treat lung cancer, but its side effects can affect patients beyond the treatment of the cancer itself. This study examined changes in different aspects of quality of life in patients who developed peripheral neuropathy during cisplatin-based chemotherapy and whether these changes differed between women and men. Quality of life was assessed before chemotherapy and after four treatment cycles using two established questionnaires. After adjustment for multiple comparisons, significant changes were observed in social/family well-being and lung cancer-specific concerns, while the other quality-of-life outcomes did not change significantly. No significant differences between women and men were found in changes over time. These findings show that different aspects of quality of life may change differently during treatment and provide a basis for further research in larger and more sex-balanced patient populations.

## 1. Introduction

Lung cancer remains a major cause of cancer-related morbidity and mortality worldwide, with a substantial proportion of patients diagnosed at an advanced stage and requiring systemic anticancer treatment [1]. Although survival remains a fundamental treatment outcome, health-related quality of life (HRQoL) has become an increasingly important component of the evaluation of patients with lung cancer [2,3]. In this population, HRQoL is multidimensional, encompassing physical and functional limitations, disease- and treatment-related symptoms, as well as emotional, social, and psychosocial aspects of well-being [2,4]. Longitudinal studies have shown that changes in specific dimensions of HRQoL may occur without corresponding changes in global quality-of-life scores [1]. The European Organisation for Research and Treatment of Cancer Quality of Life Questionnaire Core 30 (EORTC QLQ-C30) and the Functional Assessment of Cancer Therapy–Lung (FACT-L) are established patient-reported outcome instruments used to assess HRQoL in patients with lung cancer [4].

Platinum-based chemotherapy remains an important component of systemic treatment for lung cancer, but neurotoxicity represents a clinically relevant complication of treatment with neurotoxic platinum compounds [5,6]. Chemotherapy-induced peripheral neuropathy (CIPN) may impair daily functioning and quality of life (QoL) and, in a substantial proportion of affected patients, symptoms may persist beyond the period of active treatment [7,8]. A recent systematic review and meta-analysis demonstrated a substantial burden of chronic moderate-to-severe or painful CIPN, particularly among patients with lung cancer and those exposed to platinum-based chemotherapy [8]. In patients with advanced lung cancer receiving platinum-based chemotherapy, greater sensory and motor CIPN impairment has been associated with poorer neuropathy-related QoL, while poorer general QoL has been associated with anxiety, depression, and a greater number of chemotherapy cycles [5]. CIPN has also been associated with broader impairments in physical functioning, daily activities, and psychological well-being [5,7].

Among platinum agents, cisplatin is a widely established chemotherapeutic agent and is particularly relevant because of its cumulative neurotoxicity and the potential for neuropathic symptoms to continue progressing after treatment cessation [9,10]. Cisplatin-induced peripheral neuropathy is predominantly sensory, may persist over time, and has been associated with long-term functional impairment and reduced QoL [9,11].

Sex-related differences have been reported in treatment-related toxicity and patient-reported outcomes during systemic anticancer treatment [6,12,13,14]. Women have been reported to experience a higher risk of severe treatment-related adverse events than men, including among patients receiving chemotherapy [12]. Sex-related differences have also been observed for CIPN in lung cancer, with a higher incidence of high-grade CIPN in women and female sex independently associated with its occurrence [6]. Studies examining patient-reported QoL in patients with lung cancer have reported varying patterns of sex-related differences across QoL outcomes and clinical settings [13,14].

Prospective studies have reported that sex-related differences in QoL among patients with lung cancer vary across specific QoL outcomes [13,14]. Women have been reported to experience greater nausea and vomiting and hair loss, whereas men have reported poorer global QoL in the inpatient setting and greater mouth and tongue soreness in the outpatient setting [13]. In another prospective multicentre study, significant sex-related differences were limited to coughing, which was more pronounced in men, and diarrhea, which was more pronounced in women, while no significant differences were observed across the remaining QoL domains [14]. Overall, these studies reported different patterns of sex-related differences across QoL outcomes and clinical settings [13,14]. These findings do not consistently indicate poorer patient-reported QoL in women than in men.

Sex-related differences have been reported in treatment-related toxicity and CIPN [6,12], while studies of patient-reported QoL in lung cancer have shown varying patterns of sex-related differences across specific QoL outcomes and clinical settings [13,14]. Therefore, the present exploratory study aimed to evaluate longitudinal changes in QoL before and after cisplatin-based chemotherapy in patients with lung cancer using the EORTC QLQ-C30 and FACT-L, and to examine whether these outcomes and their changes over time differed between women and men.

## 2. Materials and Methods

### 2.1. Study Design and Participants

This prospective cohort study was conducted from December 2013 to January 2018 at the Clinic of Pulmonology, Clinic of Physical Medicine and Rehabilitation, and Clinic of Neurology, Clinical Centre of Serbia, Belgrade, Serbia. The original cohort included 106 adult patients with newly diagnosed, histopathologically confirmed lung cancer.

Eligible participants were patients aged >18 years with an Eastern Cooperative Oncology Group (ECOG) performance status ≤ 3. Patients with conditions that could represent alternative causes of peripheral neuropathy, including diabetes, alcohol-related intoxication, renal failure, hyperthyroidism, systemic or haematological diseases, sarcoidosis, hepatitis, HIV infection, vitamin B12 deficiency, gammopathy, previous radiotherapy, or recent trauma or surgery, were excluded.

At baseline, 68 patients had no evidence of peripheral neuropathy and subsequently received cisplatin-based chemotherapy. Neurological examination and electrophysiological assessment were performed at baseline and repeated after four cycles of chemotherapy in patients available for follow-up. During the follow-up period, eight patients did not complete the assessment after four chemotherapy cycles: four were referred for radiotherapy after the second or third cycle, and four withdrew from the study. Of the remaining 60 patients, 40 developed cisplatin-induced peripheral neuropathy, defined by the presence of neuropathic symptoms and confirmed by electrophysiological examination, whereas 20 did not develop peripheral neuropathy.

The present sex-stratified longitudinal analysis was restricted to the 40 patients who developed cisplatin-induced peripheral neuropathy and had complete QoL assessments before and after chemotherapy. The 20 patients who did not develop peripheral neuropathy were not included in this analysis.

These 40 patients were also included in a previous publication evaluating the effects of transcutaneous electrical nerve stimulation (TENS) on neuropathic pain and quality of life [11]. The present study addresses a different research question and includes only the quality-of-life assessments obtained before chemotherapy and after four cycles of cisplatin-based chemotherapy, prior to the TENS intervention. It focuses specifically on sex-stratified longitudinal changes across individual quality-of-life domains. Post-TENS outcomes are not included in the present analysis, and the sex-stratified repeated-measures analysis reported here was not presented in the previous publication.

### 2.2. Quality-of-Life Assessment

QoL was assessed using the EORTC QLQ-C30 and the FACT-L. As this was an observational study, cisplatin-based chemotherapy regimens and treatment schedules were determined by the treating oncology team as part of routine clinical care. Treatment cycles were generally scheduled at approximately 21-day intervals in accordance with standard clinical protocols, with the timing of individual cycles adjusted when clinically indicated according to the patient’s condition and treatment tolerance. All patients included in the present analysis completed four chemotherapy cycles. QoL assessments were performed before the initiation of chemotherapy and repeated after the fourth cycle, approximately four months after treatment initiation. Exact information on individual treatment intervals, any treatment delays, and the number of days between administration of the fourth cycle and the follow-up QoL assessment was not consistently documented. The EORTC QLQ-C30 consists of 30 items and comprises five functional scales—Physical Functioning (PF), Role Functioning (RF), Emotional Functioning (EF), Cognitive Functioning (CF), and Social Functioning (SF)—as well as Global Health Status/Quality of Life. The symptom scales include Fatigue (FA), Nausea and Vomiting (NV), and Pain (PA), while the single-symptom items include Dyspnea (DY), Insomnia (SL), Appetite Loss (AP), Constipation (CO), Diarrhea (DI), and Financial Difficulties (FI). Scores were transformed to a 0–100 scale, with higher scores on the functional scales and Global Health Status/Quality of Life indicating better functioning and QoL, and higher scores on the symptom scales and single items indicating greater symptom burden or problems.

The FACT-L assesses Physical Well-Being (PWB), Social/Family Well-Being (SWB), Emotional Well-Being (EWB), Functional Well-Being (FWB), and lung cancer-specific concerns using the Lung Cancer Subscale (LCS). The FACT-G Total Score, representing overall general QoL, was calculated as the sum of PWB, SWB, EWB, and FWB. The FACT-L Total Score additionally incorporates the LCS, while the FACT-L Trial Outcome Index (FACT-L TOI) comprises PWB, FWB, and LCS. Higher scores indicate better QoL. Permission to use both questionnaires was obtained.

### 2.3. Ethical Considerations

The study was conducted in accordance with the Declaration of Helsinki. Before the initial pilot and diagnostic phase commenced, written institutional authorization was granted by the Collegium of the Clinic for Pulmonology, Clinical Centre of Serbia (No. 1955/2, 6 November 2013), permitting the initial study activities and access to eligible patients with lung cancer. The approval process comprised sequential institutional and faculty-level stages. The study protocol subsequently received formal approval from the Ethics Committee of the Faculty of Medicine, University of Belgrade (protocol code 29/XII-4, 28 December 2015). Prior to inclusion, all participants received appropriate information regarding the study and provided written informed consent.

### 2.4. Statistical Analysis

Data were analyzed using descriptive and inferential statistical methods. Descriptive statistics included measures of central tendency (mean), measures of variability (standard deviation), and absolute and relative frequencies. Changes in QoL scores were analyzed using a General Linear Model (GLM) for repeated measures, with time (before vs. after chemotherapy) as the within-subject factor and sex as the between-subject factor. Main effects of time and sex, as well as the time × sex interaction, were evaluated. Parameters analyzed using this method are presented as means with 95% confidence intervals (95% CIs). To account for multiple testing across QoL domains, the Benjamini–Hochberg procedure was applied to control the false discovery rate (FDR). Adjustments were performed separately for each questionnaire and model effect (time, sex, and time × sex interaction). Statistical significance was set at FDR-adjusted *p*-value < 0.050. Statistical analyses were performed using IBM SPSS Statistics for Windows, version 26.0.

## 3. Results

### 3.1. Demographic and Clinical Characteristics

A total of 40 patients with lung cancer were included in the study. Of these, 27 (67.5%) were men and 13 (32.5%) were women. The mean age of the participants was 63.4 ± 7.5 years. Overall, 70.0% of the participants were current smokers, with a smoking history of 39.1 ± 8.9 years. Most participants were married (85.0%), had completed secondary education (75.0%), and were retired (47.5%). The majority lived with family members (95.0%). Regarding disease characteristics, among the 39 patients with available staging data, most patients were diagnosed with stage III (46.2%) or stage IV (48.7%) disease, while only two patients had stage I or II disease. Non-small-cell lung cancer was the predominant histological type (72.5%), whereas small-cell lung cancer was present in 27.5% of the study population. Detailed sociodemographic and clinical characteristics of the study population are presented in Table 1.

### 3.2. Changes in EORTC QLQ-C30 Scores During Chemotherapy

Changes in EORTC QLQ-C30 domain scores before and after chemotherapy according to sex are presented in Table 2. No significant main effects of time or sex and no significant time x sex interactions were observed for any EORTC QLQ-C30 domain (all FDR-adjusted *p* > 0.05).

### 3.3. Changes in FACT-L Scores During Chemotherapy

Table 3 summarizes the changes in FACT-L domain scores before and after chemotherapy in male and female patients. Among the analyzed FACT-L domains, significant changes over time were detected for SWB (FDR-adjusted *p* = 0.024) and the LCS (FDR-adjusted *p* = 0.044). SWB scores significantly decreased after chemotherapy, whereas LCS scores significantly increased.

**Figure 1:**
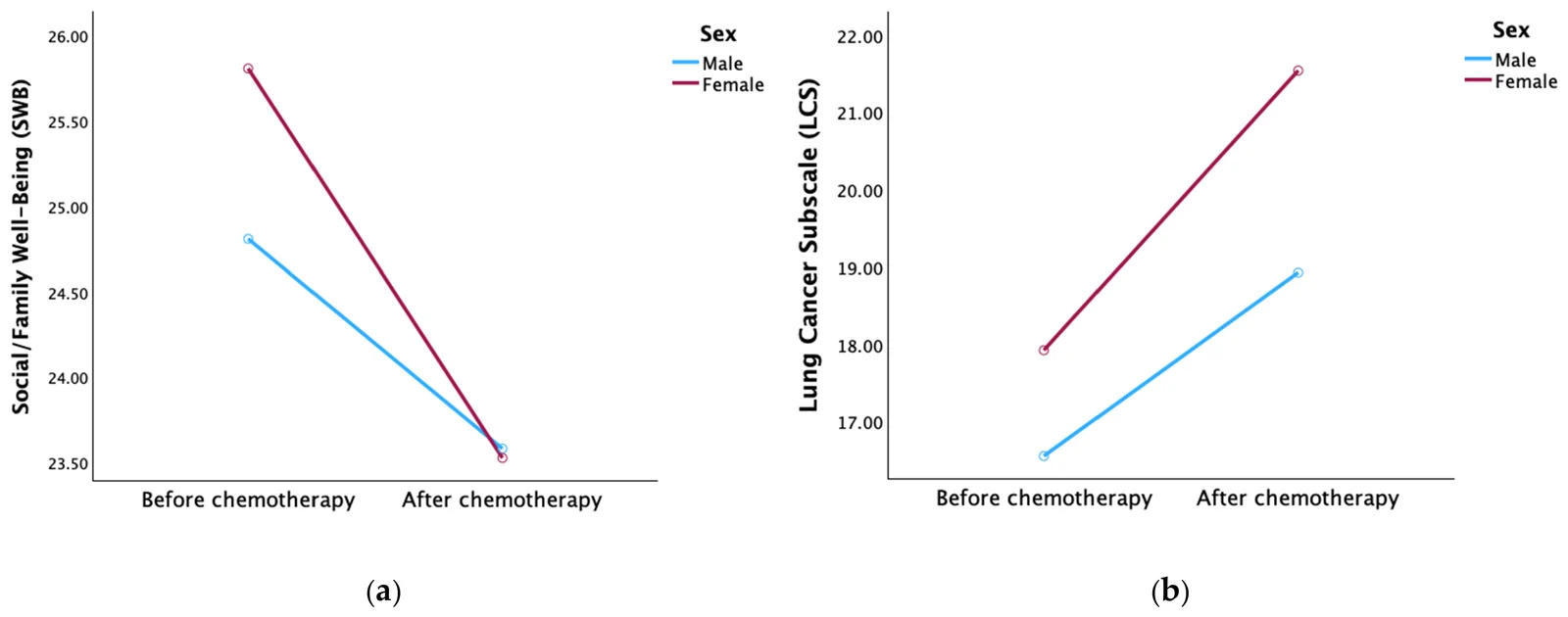
Changes in mean FACT-L scores for (**a**) Social/Family Well-Being (SWB) and (**b**) Lung Cancer Subscale (LCS) before and after chemotherapy according to sex.

Figure 1 illustrates the temporal changes observed in the SWB and LCS domains. In contrast, PWB, FWB, EWB, the FACT-L TOI, FACT-G Total Score, and FACT-L Total Score showed no significant changes over time (all FDR-adjusted *p* > 0.05). No significant main effects of sex or time × sex interactions were identified for any FACT-L domain (all FDR-adjusted *p* > 0.05) (Table 3).

## 4. Discussion

The present study evaluated longitudinal changes in QoL before and after cisplatin-based chemotherapy in patients with lung cancer who developed cisplatin-induced peripheral neuropathy, with particular attention to differences according to sex. The principal findings were that, after adjustment for multiple testing, none of the EORTC QLQ-C30 domains changed significantly over time. On the FACT-L, SWB decreased significantly, whereas LCS scores increased significantly. No significant main effect of sex or time × sex interaction was identified for any EORTC QLQ-C30 or FACT-L domain. Taken together, these findings suggest different patterns of change across specific QoL outcomes during chemotherapy.

The absence of significant changes in most EORTC QLQ-C30 domains in the present study is consistent with earlier findings by Wintner et al., who prospectively followed patients with lung cancer during different lines of chemotherapy and found that QoL scores remained largely unchanged during treatment [1]. None of the calculated monthly changes in that study reached established thresholds for clinical significance, despite differences in QoL across treatment lines. More recent studies have assessed CIPN alongside multiple dimensions of patient-reported QoL [5,15]. In patients with advanced lung cancer receiving platinum-based chemotherapy, greater sensory and motor CIPN impairment was associated with poorer neuropathy-related QoL, whereas poorer general QoL was associated with anxiety, depression, and a greater number of chemotherapy cycles [5]. Systematic reviews of patient-reported outcome measures have described instruments assessing different dimensions of CIPN-related and broader QoL [4,15]. In the present cohort, Global Health Status and most functional and symptom outcomes did not change significantly between the assessments before chemotherapy and after four treatment cycles following adjustment for multiple testing.

The FACT-L findings showed different patterns of change across QoL outcomes during chemotherapy. After adjustment for multiple testing, SWB decreased significantly, whereas LCS scores increased significantly. Psychosocial burden is common among patients with lung cancer and may coexist with substantial symptom burden and impaired global QoL [2]. In the study by Koch et al., almost half of patients screened positive for a need for psychosocial support, and psychosocial distress was related to fatigue, existential fear, and poorer global QoL [2]. In the present cohort, the significant decrease in SWB indicates poorer self-reported SWB after chemotherapy. The FACT-L assesses general dimensions of well-being as well as lung cancer-specific concerns [4].

After adjustment for multiple testing, no significant time × sex interactions were identified for any EORTC QLQ-C30 or FACT-L outcome. Previous studies have reported varying patterns of sex-related differences across QoL outcomes in patients with lung cancer [13,14]. In a prospective multicentre study of 200 patients, Koch et al. found no significant sex-related differences in most QoL domains, with significant differences limited to coughing and diarrhea [14]. In a later study, sex-related differences varied according to treatment setting, with women reporting greater nausea/vomiting and hair loss in the inpatient setting, whereas men reported poorer global QoL; among outpatients, men reported more mouth and tongue soreness [13]. Together, these studies reported different patterns of sex-related differences across specific QoL outcomes and clinical settings [13,14].

The absence of significant time × sex interactions in QoL outcomes should be considered separately from previously reported sex-related differences in treatment toxicity. In a pooled analysis of more than 23,000 patients enrolled in cancer clinical trials, women had a higher risk of severe treatment-related adverse events than men, including a higher risk of severe symptomatic adverse events [12]. Sex-related differences have also been demonstrated specifically for CIPN in patients with lung cancer [6]. In the analysis by von Itzstein et al., grade ≥ 3 CIPN occurred more frequently in women than in men, and female sex remained independently associated with high-grade CIPN after multivariable adjustment. Higher age, greater BMI, greater chemotherapy exposure, and regimens containing higher doses of cisplatin or paclitaxel were also associated with high-grade neuropathy. However, more recent evidence has not consistently confirmed sex-related differences in all CIPN outcomes. In a propensity score-matched analysis, Argyriou et al. found no significant differences between women and men in the incidence or severity of chemotherapy-induced neuropathic pain, although older men had a greater likelihood of severe pain at the end of chemotherapy [16]. Taken together, these findings indicate that sex-related differences in treatment toxicity and CIPN are not uniform and may vary according to the population and outcome examined [6,12,16], while studies of patient-reported QoL have likewise reported varying patterns across specific QoL outcomes [13,14].

Our findings should also be interpreted in the context of the particular neurotoxicity associated with cisplatin. Cisplatin is associated with cumulative peripheral neurotoxicity, and susceptibility to severe neurotoxic effects has been related to cumulative dose and patient characteristics [9]. Cisplatin-induced neuropathy may continue to progress after treatment cessation, a phenomenon commonly described as coasting [10]. Differences in the clinical course of peripheral neuropathy have been reported between cisplatin and other neurotoxic chemotherapy agents, including paclitaxel. Chronic CIPN may substantially affect physical function, daily activities, psychological well-being, and long-term QoL [7]. A recent systematic review and meta-analysis found a substantial prevalence of chronic moderate-to-severe or painful neuropathy among patients with established CIPN, with particularly high estimates among patients with lung cancer and those exposed to platinum-based chemotherapy [8]. Chronic CIPN may persist beyond the period of active chemotherapy and affect patients after treatment completion [7,8].

The EORTC QLQ-C30 and FACT-L assess multiple aspects of QoL, including functioning, symptom burden, well-being, and lung cancer-specific concerns [4]. However, statistically significant changes in patient-reported outcomes do not necessarily indicate clinically meaningful changes, and thresholds for meaningful change may vary according to the instrument, domain, direction of change, and assessment time point [17,18]. In patients with lung cancer, minimally important differences for the EORTC QLQ-C30 have been shown to vary across domains and according to whether improvement or deterioration is being evaluated [18].

Beyond the direction of individual scores, the decrease in SWB highlights the psychosocial dimension of CIPN and cancer treatment. In patients with CIPN, psychosocial adaptation has been associated with neuropathy severity, motor and autonomic symptoms, disease stage, education, employment status, and caregiver availability [19]. Greater chemotherapy-induced neurotoxicity has also been independently associated with depression, anxiety, psychological distress, and poorer sleep [20]. Studies of coping during cancer treatment indicate that women and men may differ in their use of engagement and disengagement strategies [21], while a small study of patients with non-metastatic lung cancer found sex-specific patterns of coping, depression, and anxiety after surgery [22]. In a mixed-cancer cohort assessed with FACT/GOG-NTX, women reported lower SWB and FWB than men, although the predominantly female sample and cross-sectional design limit direct comparison with the present findings [23]. Together, these studies support a multidimensional interpretation of SWB and caution against assuming a uniform direction of sex-related differences.

The potential clinical implications extend beyond symptom scores. Qualitative data show that CIPN can compromise dexterity, everyday activities, social interactions, and patients’ sense of independence [24], while persistent CIPN has been associated with impaired QoL months after chemotherapy [25]. Although the present study did not evaluate treatment, systematic-review evidence suggests that physiotherapy interventions may improve QoL, strength, balance, pain, and neuropathy-related outcomes, albeit with substantial heterogeneity and generally small samples [26]. In patients with advanced lung cancer receiving chemotherapy, a randomized exercise study found that a 12-week intervention mitigated worsening of peripheral neuropathy and reduced pain [27]. Recent randomized studies of home-based electrotherapy and wearable TENS have also reported improvements or promising preliminary effects in selected CIPN outcomes, while emphasizing the need for larger confirmatory trials [28,29].

Several limitations should be considered when interpreting these findings. The sample size was small, particularly among women, which may have limited the ability to detect sex-related differences and time × sex interactions. Therefore, nonsignificant interaction effects should not be interpreted as evidence of equivalence between women and men. The cohort was restricted to patients who developed cisplatin-induced peripheral neuropathy and did not include the 20 patients who completed four chemotherapy cycles without developing neuropathy. This restriction may limit the generalizability of the findings to all patients with lung cancer receiving cisplatin-based chemotherapy and prevents determination of whether the observed QoL changes were specific to neuropathy or reflected the broader effects of chemotherapy, the course of lung cancer, or other clinical factors. In addition, the analysis did not adjust for potentially relevant clinical and sociodemographic factors. Previous studies have identified age, BMI, chemotherapy regimen, cumulative treatment exposure, psychological factors, and socioeconomic characteristics as factors associated with neurotoxicity or QoL outcomes [5,6,30,31]. Finally, QoL was assessed at only two time points, before chemotherapy and after four cycles. Longer follow-up would be needed to determine the subsequent course of the observed QoL changes, particularly given the potential persistence and delayed progression of CIPN [7,8,10].

Despite these limitations, the study provides a longitudinal assessment of multiple QoL outcomes in a clinically defined cohort of patients with lung cancer who developed cisplatin-induced peripheral neuropathy. After adjustment for multiple testing, no statistically significant changes were identified in the EORTC QLQ-C30 domains. On the FACT-L, SWB decreased significantly, whereas LCS scores increased significantly, while the remaining outcomes did not change significantly. No statistically significant main effects of sex or time × sex interactions were identified for any EORTC QLQ-C30 or FACT-L outcome. However, given the limited and sex-imbalanced sample, these findings do not allow firm conclusions regarding sex-related differences in longitudinal QoL changes. Larger prospective studies with longer follow-up and more balanced samples of women and men are needed to determine whether sex modifies longitudinal changes in specific QoL outcomes during and after cisplatin-based chemotherapy.

## 5. Conclusions

In conclusion, among patients with lung cancer who developed cisplatin-induced peripheral neuropathy, no EORTC QLQ-C30 domain showed a statistically significant change after adjustment for multiple testing. On the FACT-L, SWB decreased significantly, whereas LCS scores increased significantly after four cycles of cisplatin-based chemotherapy. No statistically significant main effects of sex or time × sex interactions were identified. Given the small and sex-imbalanced sample, these findings should be interpreted cautiously and confirmed in larger prospective studies.

## Figures and Tables

**Table 1:** Sociodemographic and clinical characteristics of the study population.

Characteristic	Total (*n* = 40)
**Age** (years), mean ± SD	63.4 ± 7.5
**Sex**	
Male	27 (67.5)
Female	13 (32.5)
**Smoking status**	
Current smoker	28 (70.0)
Former smoker	12 (30.0)
**Smoking history (years),** mean ± sd	39.1 ± 8.9
**Marital status**	
Married	34 (85.0)
Widowed	2 (5.0)
Divorced	1 (2.5)
Never married	3 (7.5)
**Education**	
Primary education	4 (10.0)
Secondary education	30 (75.0)
Post-secondary education	3 (7.5)
University education	3 (7.5)
**Employment status**	
Unemployed	8 (20.0)
Employed	13 (32.5)
Retired	19 (47.5)
**Living arrangement**	
Living alone	2 (5.0)
Living with family	38 (95.0)
**Histological type**	
NSCLC	29 (72.5)
SCLC	11 (27.5)
**Disease stage (*n* = 39)**	
Stage I–II	2 (5.1)
Stage III	18 (46.2)
Stage IV	19 (48.7)

Data are presented as n (%) unless otherwise indicated. Stage data were unavailable for one participant; percentages for disease stage were calculated using the available data (n = 39). SD = standard deviation; NSCLC = non-small-cell lung cancer; SCLC = small-cell lung cancer.

**Table 2:** EORTC QLQ-C30 domain scores before and after chemotherapy according to sex.

Domain		Male (*n* = 27) ^a^	Female (*n* = 13) ^a^	Time *p*	Sex *p*	Time × Sex *p*
**Functional Scales**						
PF	Before	76.0 (68.8–83.3)	70.8 (55.0–86.5)	0.987	0.884	0.485
	After	69.9 (61.9–77.8)	79.5 (69.8–89.1)			
RF	Before	69.1 (60.2–78.0)	64.1 (41.6–86.6)	0.987	0.884	0.485
	After	59.3 (46.7–71.8)	74.4 (57.6–91.1)			
EF	Before	76.5 (69.2–83.9)	70.5 (48.6–92.4)	0.987	0.884	0.886
	After	76.5 (68.0–85.1)	73.7 (60.1–87.3)			
CF	Before	93.2 (87.1–99.3)	91.0 (82.2–99.9)	0.987	0.884	0.521
	After	88.3 (79.9–96.6)	94.9 (83.7–106.0)			
SF	Before	86.4 (79.3–93.5)	78.2 (59.2–97.2)	0.390	0.884	0.886
	After	74.7 (64.8–84.6)	69.2 (54.5–84.0)			
**Global Health Status**						
QL	Before	57.1 (48.6–65.6)	59.6 (47.8–71.4)	0.987	0.884	0.886
	After	56.2 (51.5–60.8)	56.4 (47.4–65.4)			
**Symptom Scales**						
FA	Before	35.8 (24.0–47.6)	37.6 (19.3–55.9)	0.987	0.884	0.733
	After	35.4 (26.8–44.0)	29.1 (17.3–40.9)			
NV	Before	4.3 (−1.3–10.0)	11.5 (−2.3–25.4)	0.987	0.884	0.521
	After	11.7 (2.6–20.8)	7.7 (−4.4–19.8)			
PA	Before	20.4 (10.0–30.8)	30.8 (9.5–52.1)	0.528	0.965	0.485
	After	37.7 (27.1–48.3)	28.2 (15.0–41.5)			
**Single-Symptom Items**						
DY	Before	23.5 (10.4–36.5)	20.5 (−2.1–43.1)	0.510	0.884	0.977
	After	13.6 (3.7–23.4)	10.3 (0.6–19.9)			
SL	Before	25.9 (12.6–39.3)	20.5 (1.2–39.9)	0.987	0.884	0.733
	After	30.9 (18.2–43.5)	15.4 (4.9–25.8)			
AP	Before	23.5 (9.9–37.0)	28.2 (6.7–49.7)	0.987	0.884	0.521
	After	29.6 (14.8–44.4)	15.4 (4.9–25.8)			
CO	Before	13.6 (3.1–24.1)	7.7 (−4.4–19.8)	0.987	0.965	0.521
	After	7.4 (0.7–14.1)	12.8 (2.6–23.0)			
DI	Before	3.7 (−0.5–7.9)	5.1 (−2.4–12.7)	0.390	0.884	0.886
	After	9.9 (2.7–17.0)	12.8 (−0.3–25.9)			
FI	Before	21.0 (9.9–32.0)	23.1 (0.7–45.4)	0.390	0.884	0.791
	After	32.1 (18.7–45.5)	43.6 (24.5–62.7)			

^a^ Data are presented as means with 95% confidence intervals (CIs); PF = Physical Functioning; RF = Role Functioning; EF = Emotional Functioning; CF = Cognitive Functioning; SF = Social Functioning; QL = Global Health Status/Quality of Life; FA = Fatigue; NV = Nausea and Vomiting; PA = Pain; DY = Dyspnea; SL = Insomnia; AP = Appetite Loss; CO = Constipation; DI = Diarrhea; FI = Financial Difficulties. *p*-values were adjusted for multiple testing using the Benjamini–Hochberg procedure.

**Table 3:** Changes in FACT-L domain scores before and after chemotherapy according to sex.

Domain		Male (*n* = 27) ^a^	Female (*n* = 13) ^a^	Time *p*	Sex *p*	Time × Sex *p*
**General Well-Being Scales**						
PWB	Before	22.2 (20.5–23.9)	20.8 (18.1–23.6)	0.256	0.951	0.483
	After	19.1 (17.2–21.1)	20.7 (16.6–24.8)			
SWB	Before	24.8 (23.6–26.1)	25.8 (23.6–28.0)	0.024 *	0.727	0.483
	After	23.6 (22.7–24.5)	23.5 (22.3–24.7)			
EWB	Before	5.3 (3.7–6.9)	5.1 (2.3–7.8)	0.693	0.904	0.924
	After	5.8 (4.7–6.9)	5.5 (3.0–8.0)			
FWB	Before	16.4 (14.3–18.6)	19.0 (15.4–22.6)	0.656	0.096	0.483
	After	14.0 (11.4–16.7)	19.2 (16.2–22.2)			
**Lung Cancer Subscale**						
LCS	Before	16.6 (14.5–18.6)	17.9 (14.6–21.2)	0.044 *	0.269	0.662
	After	18.9 (16.5–21.4)	21.5 (20.0–23.1)			
**Summary Scores**						
FACT-L TOI	Before	55.2 (50.3–60.1)	57.8 (49.6–65.9)	0.934	0.269	0.483
	After	52.1 (46.1–58.1)	61.4 (53.4–69.3)			
FACT-G Total	Before	68.8 (65.2–72.3)	70.7 (65.9–75.5)	0.205	0.269	0.483
	After	62.6 (58.1–67.1)	68.8 (60.0–77.7)			
FACT-L Total	Before	85.3 (80.5–90.1)	88.7 (81.6–95.7)	0.832	0.269	0.483
	After	81.5 (75.3–87.8)	90.4 (80.4–100.3)			

^a^ Data are presented as means (95% CIs); PWB = Physical Well-Being; SWB = Social/Family Well-Being; EWB = Emotional Well-Being; FWB = Functional Well-Being; LCS = Lung Cancer Subscale; FACT-L TOI = Functional Assessment of Cancer Therapy–Lung Trial Outcome Index; FACT-G = Functional Assessment of Cancer Therapy–General; FACT-L = Functional Assessment of Cancer Therapy–Lung. *p*-values were adjusted for multiple testing using the Benjamini–Hochberg procedure; * FDR-adjusted *p* < 0.05.

## Data Availability

The anonymized minimal dataset supporting the findings of this study is available from the corresponding author upon reasonable request. Individual-level clinical data are not publicly available due to ethical restrictions and the need to protect participant privacy and confidentiality.

## Abbreviations

The following abbreviations are used in this manuscript:

CIPN	Chemotherapy-Induced Peripheral Neuropathy
QoL	Quality of Life
HRQoL	Health-Related Quality of Life
EORTC QLQ-C30	European Organisation for Research and Treatment of Cancer Quality of Life Questionnaire Core 30
FACT-L	Functional Assessment of Cancer Therapy–Lung
FACT-G	Functional Assessment of Cancer Therapy–General
ECOG	Eastern Cooperative Oncology Group
NSCLC	Non-Small-Cell Lung Cancer
SCLC	Small-Cell Lung Cancer
PF	Physical Functioning
RF	Role Functioning
EF	Emotional Functioning
CF	Cognitive Functioning
SF	Social Functioning
QL	Global Health Status/Quality of Life
FA	Fatigue
NV	Nausea and Vomiting
PA	Pain
DY	Dyspnea
SL	Insomnia
AP	Appetite Loss
CO	Constipation
DI	Diarrhea
FI	Financial Difficulties
PWB	Physical Well-Being
SWB	Social/Family Well-Being
EWB	Emotional Well-Being
FWB	Functional Well-Being
LCS	Lung Cancer Subscale
FACT-L TOI	Functional Assessment of Cancer Therapy–Lung Trial Outcome Index
GLM	General Linear Model
CI	Confidence Interval
